# The origin of neocortical nitric oxide synthase-expressing inhibitory neurons

**DOI:** 10.3389/fncir.2012.00044

**Published:** 2012-07-09

**Authors:** Xavier H. Jaglin, Jens Hjerling-Leffler, Gord Fishell, Renata Batista-Brito

**Affiliations:** ^1^NYU Neuroscience Institute, New York University Langone Medical CenterNew York, NY, USA; ^2^Division of Molecular Neurobiology, Department of Medical Biochemistry and Biophysics, Karolinska InstituteStockholm, Sweden; ^3^Departments of Cell Biology and Neural Science, New York University Langone Medical CenterNew York, NY, USA

**Keywords:** inhibition, interneuron, cortex, hippocampus, nNOS, GABAergic, fate mapping, MGE

## Abstract

Inhibitory neurons are critical for regulating effective transfer of sensory information and network stability. The precision of inhibitory function likely derives from the existence of a variety of interneuron subtypes. Their specification is largely dependent on the locale of origin of interneuron progenitors. Neocortical and hippocampal inhibitory neurons originate the subpallium, namely in the medial and caudal ganglionic eminences (MGE and CGE), and in the preoptic area (POA). In the hippocampus, neuronal nitric oxide synthase (nNOS)-expressing cells constitute a numerically large GABAergic interneuron population. On the contrary, nNOS-expressing inhibitory neurons constitute the smallest of the known neocortical GABAergic neuronal subtypes. The origins of most neocortical GABAergic neuron subtypes have been thoroughly investigated, however, very little is known about the origin of, or the genetic programs underlying the development of nNOS neurons. Here, we show that the vast majority of neocortical nNOS-expressing neurons arise from the MGE rather than the CGE. Regarding their molecular signature, virtually all neocortical nNOS neurons co-express the neuropeptides somatostatin (SST) and neuropeptide Y (NPY), and about half of them express the calcium-binding protein calretinin (CR). nNOS neurons thus constitute a small cohort of the MGE-derived SST-expressing population of cortical inhibitory neurons. Finally, we show that conditional removal of the transcription factor *Sox6* in MGE-derived GABAergic cortical neurons results in an absence of SST and CR expression, as well as reduced expression of nNOS in neocortical nNOS neurons. Based on their respective abundance, origin and molecular signature, our results suggest that neocortical and hippocampal nNOS GABAergic neurons likely subserve different functions and have very different physiological relevance in these two cortical structures.

## Introduction

Brain activity is regulated by the interaction of two major types of neural cells: excitatory neurons that use the neurotransmitter glutamate and inhibitory neurons that use the neurotransmitter γ-aminobutyric acid (GABA). Inhibitory neurons are critical for maintaining the excitatory-inhibitory balance necessary for the effective transfer of information while preventing runaway excitation, and consequently play important roles in regulating network activity (Pouille and Scanziani, 2001; Markram et al., 2004; Haider and McCormick, 2009). Severe disruption of GABAergic inhibition profoundly alters neocortical activity patterns and leads to seizures, while milder changes in inhibitory neuron number and function are strongly linked to psychiatric disorders, such as schizophrenia and autism (Volk et al., 2002; Yau et al., 2003; Belmonte et al., 2004; Levitt et al., 2004; Cossart et al., 2005; Dani et al., 2005; Caceda et al., 2007; Gonzalez-Burgos and Lewis, 2008; Morris et al., 2008). Inhibition is thus hypothesized to be a critical regulator of normal brain function and a key cause of dysfunction in disease. Inhibitory function relies on the existence of a variety of GABAergic inhibitory neuronal subtypes. Most inhibitory neurons display locally projecting axons and have been consequently named interneurons. Besides this morphological variety, different classes of inhibitory neurons exhibit distinct intrinsic membrane properties, molecular markers, connectivity, and synaptic specializations, and therefore likely mediate specific roles within cortical circuits (Fishell and Rudy, 2011). In the murine system, although many characteristics are not acquired until several weeks postnatally, the specification of GABAergic neurons in the mature animal is largely established during embryonic development, through the function of specific transcription factors exclusive to different spatial regions (Flames et al., 2004; Butt et al., 2005, 2008; Wonders and Anderson, 2006; Fogarty et al., 2007; Wonders et al., 2008; Batista-Brito and Fishell, 2009; Gelman et al., 2009).

While the majority of neocortical GABAergic neurons project a highly ramified axon locally, these interneurons are not the sole source of inhibition within the neocortex. A small portion of GABAergic neurons projects axons to distant neocortical areas, including regions in the ipsilateral hemisphere and contralateral hemispheres (McDonald and Burkhalter, 1993; Gonchar et al., 1995; Aroniadou-Anderjaska and Keller, 1996; Chowdhury et al., 1996; Salin and Prince, 1996; Kimura and Baughman, 1997). A study combining labeling of GABAergic projection neurons, retrograde labeling and axon tracing methods revealed that the vast majority of neocortical long-range projecting GABAergic neurons belong to a group of cells expressing the neuronal isoform of nitric oxide synthase (nNOS) (Tomioka et al., 2005). This result suggests that even though low in number (Gonchar et al., 2007), neocortical nNOS GABAergic neurons have the potential to strongly influence neocortical networks. Most of the studies on the origin and function of nNOS cells have focused on the hippocampus where nNOS is expressed in a numerically large population of GABAergic neurons (Fuentealba et al., 2008; Tricoire et al., 2010, 2011). However, the function and origin of nNOS-expressing inhibitory neurons in the neocortex remain largely unexplored.

Neocortical and hippocampal GABAergic inhibitory neurons are produced in the neurogenic ganglionic eminences of the ventral telencephalon (or subpallium) and migrate tangentially toward the neocortex and hippocampus (Anderson et al., 1997a; Lavdas et al., 1999; Sussel et al., 1999; Pleasure et al., 2000; Marin and Rubenstein, 2001; Wichterle et al., 2001). The ventral ganglionic eminences express numerous genes known to be essential for the generation of GABAergic cells. These include genes with widespread expression throughout the subpallium, such as the *Distaless homeobox* (*Dlx*) family of genes (*Dlx1*, *2*, *5*, and *6*) (Anderson et al., 1997a,b; Cobos et al., 2005; Ghanem et al., 2007; Potter et al., 2009), which have been shown to be critical for interneuron migration and specification (Anderson et al., 1997b; Pleasure et al., 2000; Petryniak et al., 2007). Most neocortical and hippocampal GABAergic neurons are produced in the embryonic proliferative regions named the medial and caudal ganglionic eminences (MGE and CGE, respectively) (Wichterle et al., 2001; Nery et al., 2002; Xu et al., 2004, 2008; Butt et al., 2005; Flames et al., 2007; Fogarty et al., 2007; Miyoshi et al., 2010; Tricoire et al., 2011), and a smaller percentage are produced in the embryonic preoptic area (POA) (Gelman et al., 2009, 2011).

In the neocortex, near all parvalbumin (PV) and somatostatin (SST) expressing inhibitory neuron subtypes are produced in the MGE (Wichterle et al., 1999; Butt et al., 2005; Fogarty et al., 2007; Wonders et al., 2008; Xu et al., 2008). The genetic cascade necessary for proper specification, differentiation, and development of MGE-derived interneurons is starting to be elucidated. *Nkx2.1* has been shown to direct the MGE-subtype specification and *Lhx6* is necessary for the proper migration and maturation of both PV- and SST-expressing interneurons (Sussel et al., 1999; Liodis et al., 2007). The transcription factor *Sox6*, which is also expressed in MGE-derived interneuron lineages (Azim et al., 2009; Batista-Brito et al., 2009), acts downstream of *Lhx6* and is required for the positioning and maturation of PV cells, and to a lesser extent, SST cells (Batista-Brito et al., 2009).

In addition to the MGE, the CGE is the second largest source of neocortical inhibitory neurons, contributing approximately 30% of all GABAergic neurons (Lee et al., 2010). Recently, it has been shown that all CGE-derived neocortical interneurons specifically express the serotonin receptor 3a (5-Ht3a) (Lee et al., 2010; Vucurovic et al., 2010), while the transcription factors CoupTF1/2 are widely but not selectively expressed within the CGE (Sousa et al., 2009). The 5-Ht3a-expressing CGE-derived GABAergic neocortical interneurons, includes the entire vasoactive intestinal peptide (VIP) and cholecystokinin (CCK)-expressing population as well as the entire SST-negative populations of calretinin (CR) and Reelin (RLN)-expressing interneurons (Nery et al., 2002; Lee et al., 2010; Miyoshi et al., 2010).

It has been recently shown that different parts of the POA selectively express *Nkx5.1* and *Dbx1*, respectively, and produce a small and highly diverse fraction of neocortical GABAergic interneurons (Gelman et al., 2009, 2011). The interneurons types produced in the *Dbx1* population are largely overlapping with the MGE-derived subtypes while it is possible that the *Nkx5.1* expressing population is included in the population expressing *5-Ht3a* (Gelman et al., 2009, 2011; Lee et al., 2010).

Similarly to neocortical interneurons, PV- and SST-expressing hippocampal interneurons originate in the MGE, whereas hippocampal interneurons expressing CCK, CR, and VIP are produced in the CGE (Tricoire et al., 2011; Keimpema et al., 2012). Neuropeptide Y (NPY) expressing hippocampal interneurons encompass a mixed repertoire of subtypes originating from the MGE, CGE and the POA (Gelman et al., 2009). Despite largely originating in the MGE, the majority of nNOS-expressing hippocampal GABAergic neuron subpopulations do not overlap with PV- or SST-expressing interneurons (Fuentealba et al., 2008; Tricoire et al., 2010, 2011), and few nNOS-positive cells co-express CR (Jinno and Kosaka, 2002a). In the hippocampus, nNOS is expressed in the majority of neurogliaform cells (NGC) and Ivy cells (IvCs) interneuron subtypes (Fuentealba et al., 2008; Tricoire et al., 2010). Within the hippocampus, nNOS colocalizes with a variety of markers (Fuentealba et al., 2008; Szabadics and Soltesz, 2009; Tricoire et al., 2010) and has been reported to be the numerically largest interneuron population (Fuentealba et al., 2008). It remains unknown if there are any homologs of the nNOS-expressing neurogliaform and Ivy interneurons within the neocortex.

Here we show that contrary to the hippocampus, nNOS-expressing cells constitute a small minority of the total neocortical GABAergic neurons. In order to investigate the developmental origin of nNOS-expressing cells, we did genetic fate mapping using cre-drivers specific for different domains of the MGE (*Nkx2.1*^*Cre*^ and *Lhx6*^*Cre*^) as well as the *5-Ht3a*^*EGFP*^-reporter line that labels the CGE-derived lineage. Our findings suggest that nNOS-expressing neocortical GABAergic neurons originate from the MGE, mainly from the most dorsal domain of the MGE (dMGE). In the hippocampus, similarly to what has been previously shown, we observed that a majority of the nNOS interneurons are derived from the MGE, but a significant portion originate within the CGE and/or POA (Tricoire et al., 2010). Both neocortical and hippocampal nNOS-expressing cells also express NPY, but in contrast to the hippocampus virtually all neocortical nNOS neurons express SST, and about half express CR. Finally, we show that the transcription factor *Sox6* has a role in the differentiation of neocortical nNOS cells, since loss of *Sox6* leads to a total absence of SST expression in neocortical nNOS cells, and possibly stunts the development of neurites.

Our results show that nNOS-expressing neocortical and hippocampal inhibitory neurons have different origins, suggesting that neocortical and hippocampal nNOS-expressing cells constitute an example of unrelated subtypes, both having acquired/retained the expression of nNOS. Understanding the functional role of this sparse population of neocortical long-range projection inhibitory neurons and the relevance of the nitrinergic signaling in cortical circuits are questions of considerable interest.

## Materials and methods

### Mouse lines

All animal handling and maintenance were performed according to the regulations of the Institutional Animal Care and Use Committee of the NYU School of Medicine. The *Dlx6*^*Cre*^ (GENSAT project at Rockefeller University), *Lhx6*^*Cre*^ (Fogarty et al., 2007), *Nkx2.1*^*Cre*^ (Xu et al., 2008), *SST*^*Cre*^ (Taniguchi et al., 2011), *5-Ht3a*^*EGFP*^ (GENSAT project at Rockefeller University), *Sox6*^*F*/+^ (Dumitriu et al., 2006), and *RCE*^*EGFP*^ (Sousa et al., 2009) mouse lines were maintained in a mixed background (Swiss Webster and C57Bl/6), and genotyped as previously described (Stenman et al., 2003; Dumitriu et al., 2006; Fogarty et al., 2007; Butt et al., 2008; Xu et al., 2008; Taniguchi et al., 2011).

### *In vivo* genetic fate mapping

To perform genetic fate mapping of neocortical and hippocampal GABAergic neurons transgenic males heterozygous for the driver line *Dlx6*^*Cre*^ (GENSAT project at Rockefeller University) were crossed to female homozygous for the *RCE*^*EGFP*^ allele reporter allele (Sousa et al., 2009). To fate map MGE-derived lineages, males heterozygous for the transgenic driver lines *Lhx6*^*Cre*^ (Fogarty et al., 2007) and *Nkx2.1*^*Cre*^ (Xu et al., 2008) were crossed to female mice homozygous for the *RCE*^*EGFP*^ reporter allele (Sousa et al., 2009). Fate mapping of the CGE-derived lineage was accomplished by using the BAC-transgenic line *5-Ht3a*^*EGFP*^ (GENSAT project at Rockefeller University).

### *In vivo Sox6* conditional loss of function

Male *Sox6*^*F*/+^; *Lhx6*^*Cre*^ or *Sox6*^*F*/+^; *SST*^*Cre*^ mice were crossed to *Sox6*^*F/F*^; *RCE*^*EGFP/EGFP*^ females to generate productive *Sox6*^*F*/+^; *Lhx6*^*Cre*^; *RCE*^*EGFP*^ (control) and *Sox6*^*F/F*^; *Lhx6*^*Cre*^; *RCE*^*EGFP*^ (mutant); or *Sox6*^*F*/+^; *SST*^*Cre*^; *RCE*^*EGFP*^ (control) and *Sox6*^*F/F*^; *SST*^*Cre*^; *RCE*^*EGFP*^ (mutant) offspring.

### Tissue preparation for immunocytochemistry

The brains of juvenile mice (P21) were fixed by transcardiac perfusion of 4% paraformaldehyde (PFA)/phosphate buffered saline (PBS) solution followed by a one hour post-fixation on ice with 4% PFA/PBS solution. Brains were rinsed with PBS and cryoprotected by using 30% sucrose/PBS solution overnight at 4°C. Tissues were embedded in Tissue Tek, frozen on dry ice, and cryosectioned at 20 μm thickness.

### Immunohistochemistry

Sections for immunohistochemistry analysis were processed using 2% normal goat serum/0.1% Triton X-100 in all procedures except washing steps, where only PBS was used. Sections were blocked for 1 h, followed by incubation with the primary antibodies overnight at 4°C. Cryostat tissue sections were stained with the following primary antibodies: rabbit anti-GFP (1:1000; Molecular Probes), rat anti-GFP (1:1000, Nacalai Tesque), chicken anti-GFP (1:1000, AbCam), mouse anti-Parvalbumin (1:1000; Sigma), rat anti-SST (1:500; Chemicon), rabbit anti-NPY (1:500; Incstar), sheep anti-NPY (1:500; Chemicon), mouse anti-Reelin (1:500; MBL international), rabbit anti-nNOS (1:1000, Chemicon), mouse anti-calretinin (1:750; Chemicon). Secondary antibodies conjugated with Alexa fluoro-dyes 488, 594, or 649 (Molecular Probes) raised from the same host were then used as blocking serum and were applied for 30 min at room temperature for visualizing the signals. Nuclear counterstaining was performed with 100 ng/ml 4,6-diamidino-2-phenylindole (DAPI) solution in PBS for 1 min. Fluorescent images were captured using a cooled-CCD camera (Princeton Scientific Instruments, NJ) using Metamorph software (Universal imaging, Dwoningtown, PA). For all embryonic and postnatal stages, counting was performed on a minimum of four coronal sections from at least two animals using the image processing and analysis software ImageJ (Wayne Rasband, NIH). Neocortical analyses were performed in the somatosensory barrel cortex (S1BF). Hippocampal analyses were carried out in CA3, CA1, and dentate gyrus. To minimize counting bias we compared sections of equivalent bregma positions (from −1.5 mm to −2.0 mm relative to bregma), defined according to the Mouse Brain atlas (Franklin and Paxinos, 2001). All data were represented as mean ± SEM.

## Results

### Neocortical nNOS-expressing cells constitute a small minority of neocortical GABAergic neurons

**Figure 1:**
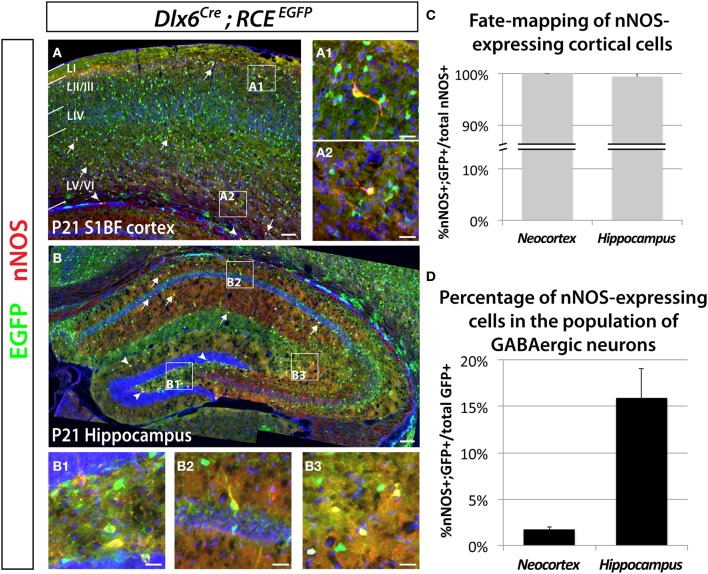
**Genetic fate mapping reveals that neocortical and hippocampal nNOS-expressing cells are GABAergic inhibitory neurons.** The inhibitory fate was verified using the pan-interneuronal driver *Dlx6*^*Cre*^ allowing the expression of the EGFP from the RCE reporter in the neocortex (somatosensory cortex) **(A)** and hippocampus **(B)**. Using this fate mapping strategy we determined the percentage of neocortical and hippocampal GABAergic neurons expressing the neuronal Nitric Oxide Synthase (nNOS) on telencephalic coronal sections. **(A)** Representative section showing sparse cells that express nNOS in the mouse barrel cortex at P21 (arrows), mainly in superficial (layer II/III) (A1) and deep (layer VI) (A2) layers as shown by the DAPI counterstaining. Note the presence of EGFP in every nNOS-expressing cell. A few nNOS-expressing neurons are also located in the white matter (arrowheads). **(B)** Scattered nNOS staining is found throughout the mouse hippocampus at P21, in all layers of the Ammon's horns CA1–3 (arrows) as well as in all layers of the dentate gyrus (arrowheads). Similar to the neocortex and irrespective of the cell's location within the hippocampus (B1, hilar region and subgranular layer of the dentate gyrus; B2, CA1 stratum pyramidale and stratum oriens; B3, CA3 stratum radiatum and stratum lacunosum-moleculare), nNOS-expressing cells are GABAergic neurons as revealed by the expression of EGFP. **(C)** Histogram showing the percentage of nNOS-expressing neurons that are fate mapped by the *Dlx6*^*Cre*^; *RCE*^*EGFP*^ line in mouse cortex and hippocampus (100 ± 0.0% and 99.5 ± 1.1%) (*n* = 3 independent brains for each genotype). **(D)** Histogram showing the percentage of *Dlx6*^*Cre*^; *RCE*^*EGFP*^ fate mapped interneurons that express nNOS in mouse cortex and hippocampus (1.7 ± 0.3% and 15.9 ± 3.2%) (*n* = 3 independent brains for each genotype). Data represent mean ± SEM. Scale bars correspond to 100 μm **(A,B)** and 25 μm (A1,A2,B1–3).

Previous work has shown that nNOS cells in the hippocampus and neocortex express markers characteristic of inhibitory neurons (Jinno et al., 2002; Gonchar et al., 2007; Fuentealba et al., 2008). In order to determine the percentage of nNOS neurons that are GABAergic, as well as the percentage of GABAergic neurons that express nNOS we performed immunostaining for nNOS in brain slices where the entire GABAergic cortical neuronal population was labeled with EGFP, using genetic fate mapping of cells expressing the pan-GABAergic gene *Dlx6* (Batista-Brito et al., 2008). Genetic fate mapping experiments using Cre/loxP technology are based on the combined use of a *driver allele* (whereby the promoter of a gene of interest drives the expression of a Cre recombinase) and a *floxed-reporter allele* (whereby the expression of the reporter gene is triggered by a recombination event mediated by the Cre recombinase). Upon recombination, the expression of the reporter gene allows for selective and cumulative labeling of cells sharing the common expression of the gene of interest at any point in their history. Here we used the *Dlx6*^*Cre*^ driver mouse line combined with the *RCE*^*EGFP*^ reporter line, whereby Cre activity removes the floxed-STOP cassette at the *Rosa* locus, resulting in permanent EGFP expression in cells that have once expressed *Dlx6.* We show that all the neocortical and hippocampal interneurons (100 ± 0.9% and 99.5 ± 1.1%, respectively) expressing nNOS also express EGFP. Therefore, we conclude that nNOS labeling in the neocortex and hippocampus is restricted to GABAergic neurons (Figures 1A–C). The number of neocortical GABAergic neurons expressing nNOS is much lower relative to the number of nNOS-expressing cells in the hippocampus (Figures 1A–C). Within the neocortex only 1.7 ± 0.3% of the total number of *Dlx6* fate mapped cells express nNOS, compared to 15.9 ± 3.2% in the hippocampus (Figure 1D). All our analyses were confined to cells that express high levels of nNOS, also known as nNOS type I neurons (Yan et al., 1996; Judas et al., 1999; Lee and Jeon, 2005). For simplicity, and because the used antibody does not allow the identification of cells expressing low levels of nNOS (type II nNOS neurons), we will refer to the cells that unambiguously express high levels of nNOS as nNOS-expressing cells throughout the manuscript. Neocortical nNOS neocortical neurons are preferentially distributed in deeper layers (V–VI), although some are located in the superficial layers (II/III) (Figure 1A). Furthermore, a few nNOS-expressing neurons are also located in the white matter. Neocortical nNOS neurons display highly elaborated processes as described elsewhere (Tomioka et al., 2005). By contrast, nNOS staining is present throughout the mouse hippocampus at P21, including all layers of the Ammon's horns CA1-3 as well as in the dentate gyrus' layers (except the granular layer) (Figure 1B).

### The majority of neocortical nNOS GABAergic neurons originate within the dMGE

In order to determine the origin of nNOS-expressing neocortical inhibitory neurons, we fate mapped neurons produced in the ventral telencephalon and located in the somatosensory cortex of P21 mice. Fate mapping of the MGE was accomplished by using the *Nkx2.1*^*Cre*^ (Xu et al., 2008) and *Lhx6*^*Cre*^ (Fogarty et al., 2007) driver lines in combination with the *RCE*^*EGFP*^ reporter allele. In *Nkx2.1*^*Cre*^; *RCE*^*EGFP*^ mice, EGFP labeled cells originate in most of the MGE (except for its most dorsal part; dMGE), and the POA (Xu et al., 2008; Sousa et al., 2009). In *Lhx6*^*Cre*^; *RCE*^*EGFP*^ mice cells originating within the entire MGE, and POA are permanently labeled with EGFP (Fogarty et al., 2007). *Lhx6* is also expressed in blood vessels, therefore, fate mapping using this line also labels blood vessels with EGFP (Fogarty et al., 2007). We labeled CGE-derived cells with the transgenic line *5-Ht3a^*EGFP*^* (Lee et al., 2010).

**Figure 2:**
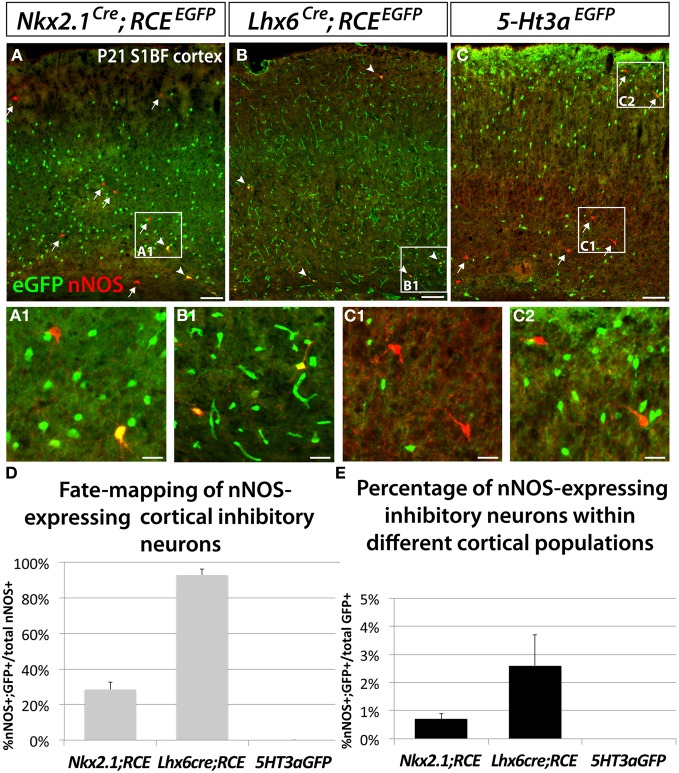
**Neocortical nNOS-expressing inhibitory neurons are derived from the medial ganglionic eminence.** To ascertain and quantify the spatial origin of cortical nNOS inhibitory neurons, we employed different fate mapping strategies based on the use of *Nkx2.1*^*Cre*^; *RCE*^*EGFP*^ **(A)**, *Lhx6*^*Cre*^; *RCE*^*EGFP*^ **(B)**, or *5-Ht3a^*EGFP*^* mice **(C). (A–C)** Representative pictures of coronal sections of P21 mouse somatosensory neocortex immunolabeled with EGFP and nNOS. Fate mapped cells expressing EGFP and nNOS are indicated by arrowheads. Arrows indicated cells expressing nNOS only. **(D,E)** Histograms showing the fate mapping of cortical nNOS GABAergic neurons and the contribution of nNOS-expressing cells to MGE and CGE populations, expressed as the percentage of nNOS+/GFP+ cells in the entire nNOS positive population **(D)**, or in the entire EGFP positive population **(E)**. The *Nkx2.1*^*Cre*^; *RCE*^*EGFP*^ fate mapping indicates that 28.5 ± 4.3% of nNOS GABAergic neurons originate from the MGE domain covered by the *Nkx2.1*^*Cre*^ driver line **(A,D)** and that nNOS cells account for 0.7 ± 0.2% of this *Nkx2.1*^*Cre*^-derived population **(A,E)** (*n* = 3 independent brains). The *Lhx6*^*Cre*^; *RCE*^*EGFP*^ and *5-Ht3a^*EGFP*^* fate mapping strategies revealed that virtually all nNOS-cINs originate from the MGE (93.0 ± 3.3%) **(B,D)** and not from the CGE (no cells co-express nNOS and GFP) **(C,D)**, respectively (*n* = 3 independent brains for each genotype). On the other hand, nNOS cells account for 2.6 ± 1.1% of MGE-derived inhibitory neurons fate mapped by the *Lhx6*^*Cre*^; *RCE*^*EGFP*^ **(B,E)**. Data represent mean ± SEM. Scale bars correspond to 100 μm **(A–C)** and 25 μm (A1,B1,C1,C2).

Our results show that nNOS neurons colocalize with EGFP in the *Nkx2.1*^*Cre*^; *RCE*^*EGFP*^ and *Lhx6*^*Cre*^; *RCE*^*EGFP*^ mice (Figures 2A,B), but not in the *5-Ht3a^*EGFP*^* animals (Figure 2C). Since *5-Ht3a^*EGFP*^* labels cells migrating out from the CGE, our results suggest that the CGE do not produce nNOS inhibitory neurons of the neocortex.

In contrast, 93 ± 3.3% of nNOS cells in the somatosensory cortex of *Lhx6*^*Cre*^; *RCE*^*EGFP*^ mice express EGFP (Figures 2B,D), leading us to conclude that the vast majority of the neocortical nNOS cells originate within the MGE. Surprisingly, only 28.5 ± 4.3% of nNOS cells also express EGFP when we used the *Nkx2.1*^*Cre*^; *RCE*^*EGFP*^ transgenic line. Comparison of the results from the *Nkx2.1*^*Cre*^; *RCE*^*EGFP*^ and *Lxh6*^*Cre*^; *RCE*^*EGFP*^ lines indicate that 70–75% of the total of nNOS cells in the somatosensory cortex originates within the dMGE, while the remaining 20–25% originates from the *Nkx2.1*^*Cre*^ domain. Moreover, this fate mapping strategy also confirmed that nNOS inhibitory neurons only account for a small portion of the MGE- and dMGE-derived cortical interneurons (respectively, 0.7 ± 0.19% of fate mapped interneurons in *Nkx2.1*^*Cre*^; *RCE*^*EGFP*^ mice and 2.6 ± 1.1% of fate mapped interneurons in *Lhx6*^*Cre*^; *RCE*^*EGFP*^ mice) (Figure 2E).

### Hippocampal nNOS GABAergic neurons do not originate within the dMGE

**Figure 3:**
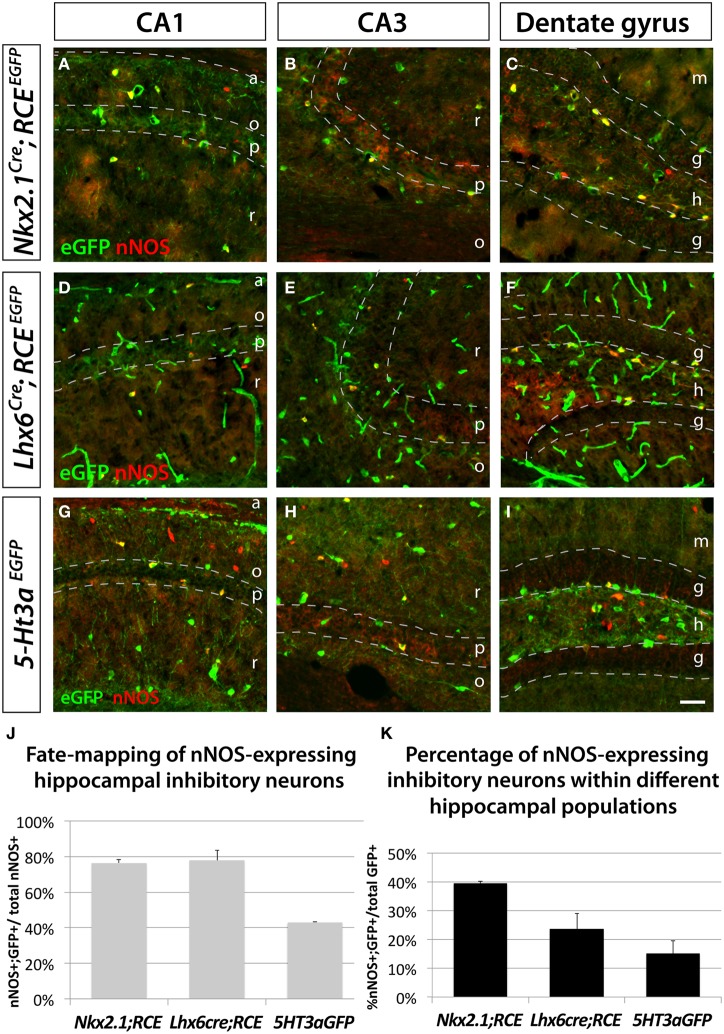
**Hippocampal nNOS-expressing inhibitory neurons are not generated from the dMGE.** To ascertain and quantify the spatial origin of nNOS inhibitory neurons scattered through the entire hippocampus (CA1–3 and dentate gyrus), we employed different fate mapping strategies based on the use of *Nkx2.1*^*Cre*^; *RCE*^*EGFP*^ **(A–C)**, *Lhx6*^*Cre*^; *RCE*^*EGFP*^ **(D–E)**, or *5-Ht3a^*EGFP*^* mice **(F–H). (A,D,G; B,E,H; C,F,I)** Representative pictures of EGFP and nNOS immunolabeled P21 mouse hippocampus that illustrate the CA1, CA3 and dentate gyrus, respectively. Although MGE-derived nNOS GABAergic neurons are found in the different layers of the CA1 and CA3 regions, they are mostly found in the hilus and subgranular layer of the dentate gyrus **(A,B,D,E)** but are absent from the granular layer **(C,F)**. Similarly, CGE-derived nNOS cells are located in the different layers of CA1, CA3 **(G,H)** and dentate gyrus, with the exception of the dentate gyrus granular layer **(I). (J,K)** Histograms showing the fate mapping of hippocampal nNOS GABAergic neurons and the contribution of nNOS-expressing cells to MGE- and CGE-derived populations of hippocampal inhibitory neurons. The *Nkx2.1*^*Cre*^; *RCE*^*EGFP*^ and *Lhx6*^*Cre*^; *RCE*^*EGFP*^ fate mappings indicates that 76.5 ± 1.9% and 77.9 ± 5.7% of nNOS GABAergic neurons originate from the MGE domains covered by the *Nkx2.1*^*Cre*^ or *Lhx6*^*Cre*^ driver lines, respectively **(J)**. Note that comparable numbers of nNOS inhibitory neurons are fate mapped by both *Nkx2.1*^*Cre*^ or *Lhx6*^*Cre*^ driver lines suggesting that the dMGE is not an origin for hippocampal nNOS inhibitory neurons. By contrast, the nNOS cells account for 39.4 ± 0.9% and 23.6 ± 5.4% of the *Nkx2.1*^*Cre*^- and *Lhx6*^*Cre*^-derived population, respectively **(K)**. The *5-Ht3a^*EGFP*^* fate mapping strategy revealed that 43.1 ± 0.4% of nNOS inhibitory neurons originate from the CGE. While conversely, nNOS GABAergic neurons account for 15.1 ± 4.5% of CGE-derived inhibitory neurons fate mapped by the *5-Ht3a^*EGFP*^*. a, alveus; o, stratum oriens; p, stratum pyramidale; r, stratum radiatum; m, molecular layer; g, granular layer; h, hilus. Data represent mean ± SEM (*n* = 3 independent brains for each genotype). Scale bar corresponds to 100 μm.

We used the fate mapping approaches previously described to test if hippocampal nNOS neurons have the same origin as their neocortical counterparts. In accordance with previous studies (Tricoire et al., 2010), we observed that the majority of hippocampal nNOS neurons are derived from the MGE, as indicated by the observation that 76.5 ± 1.9% and 77.9 ± 5.7% of nNOS inhibitory neurons coexpress EGFP in the transgenic lines *Nkx2.1*^*Cre*^; *RCE*^*EGFP*^ (Figures 3A–C,J) and *Lxh6*^*Cre*^; *RCE*^*EGFP*^ (Figures 3D–F,J), respectively. Comparison of the results from the *Nkx2.1*^*Cre*^; *RCE*^*EGFP*^ and *Lxh6*^*Cre*^; *RCE*^*EGFP*^ lines indicate that, contrary to what is observed in the neocortex, only a small minority (less than 2%) of hippocampal nNOS cells originate from the dMGE. Furthermore, we observed in the hippocampus that 43.1 ± 0.4% of nNOS neurons were co-labeled with EGFP in the transgenic *5-Ht3a^*EGFP*^* line (Figures 3G–I,J). While it has been previously recognized that the CGE produces some hippocampal nNOS neurons by using the transgenic lines *Gad65*^*GFP*^ (Tricoire et al., 2010) and *Mash1*^*CreER*^ (Tricoire et al., 2010), the contribution of the CGE towards nNOS hippocampal neurons perhaps have been underestimated. This could be due to the fact that the *Gad65*^*GFP*^ lineage does not recapitulate the totality of the CGE-derived lineage of interneurons, and due to the mosaic inducible nature of the *Mash1*^*CreER*^ line. However, the fact that 77.9 ± 5.7% (from the *Lhx6*^*Cre*^) and 43.1 ± 0.4% (from *5-Ht3a^*EGFP*^*) adds up to more than a 100% it is likely that *5-Ht3a^*EGFP*^* is less a specific CGE-marker in the hippocampus compared to the cortex. Another possible explanation, which is not mutally exclusive of these observations, is that cell arising in the POA are both EGFP-positive in the *Lhx6*^*Cre*^; *RCE*^*EGFP*^ and in the *5-Ht3a^*EGFP*^* lines in the hippocampus. If the latter is the case, up to 21% of the nNOS population could be POA-derived, a much higher number than in the cortex.

Moreover, this fate mapping analysis in the hippocampus revealed that nNOS inhibitory neurons account for 39.4 ± 0.9% of *Nkx2.1*^*Cre*^; *RCE*^*EGFP*^ fate mapped cells, 23.6 ± 5.4% of *Lhx6*^*Cre*^; *RCE*^*EGFP*^ fate mapped cells and 15.1 ± 4.5% of of *5-Ht3a*^*EGFP*^ fate mapped cells (Figure 3K).

**Figure 4:**
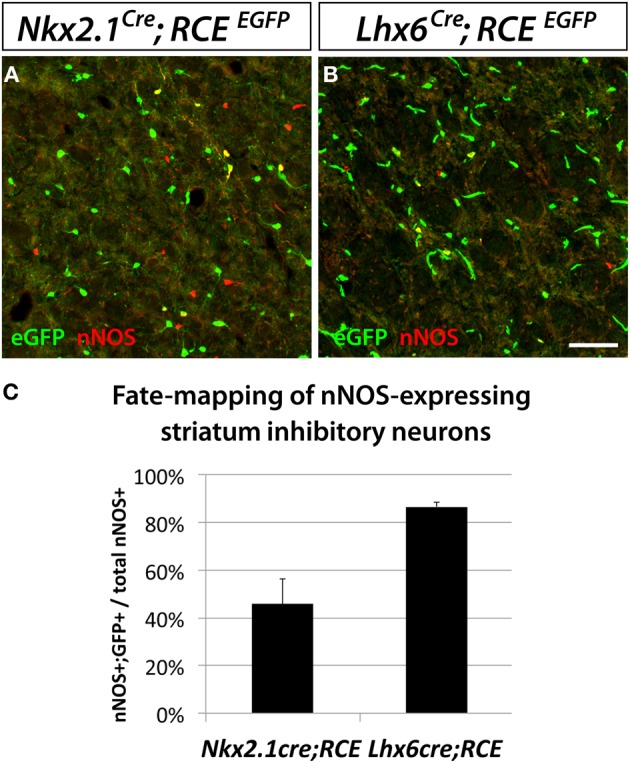
**Striatal nNOS GABAergic neurons are derived from the entire MGE.** To determine and quantify the spatial origin of nNOS GABAergic neurons present in the striatum, we performed fate mapping experiments based on the use of *Nkx2.1*^*Cre*^; *RCE*^*loxP*^ **(A)**, and *Lhx6*^*Cre*^; *RCE*^*EGFP*^ **(B)**. This set of experiments revealed that 45.9 ± 10.5% and 86.7 ± 1.7% of nNOS GABAergic neurons originate from the MGE domains covered by the *Nkx2.1*^*Cre*^ or *Lhx6*^*Cre*^ driver lines, respectively **(C)**. Data represent mean ± SEM (*n* = 3 independent brains for each genotype). Scale bar corresponds to 50 μm.

Interestingly, we observed that despite the striatum being typically thought of as a structure less related to the neocortex than the hippocampus, the origins of striatal and neocortical nNOS cells are more similar than the origins of hippocampal and neocortical nNOS cells. The vast majority of striatal nNOS cells originate from the MGE, as indicated by *Lhx6*^*Cre*^ fate mapping (Compare Figure 4A to B, Figure 4C) (Gittis et al., 2010). Similarly to the neocortex, the dMGE is a major source of striatal nNOS neurons, producing about 40% of this population (substract 45.9% of nNOS cells fate-mapped with the line *Nkx2.1*^*Cre*^; *RCE*^*EGFP*^ to 86.7% of nNOS cells fate mapped with the line *Lhx6*^*Cre*^; *RCE*^*EGFP*^) (Figure 4C).

### Neocortical nNOS neurons belong to the MGE-derived somatostatin-positive population of GABAergic inhibitory neurons

**Figure 5:**
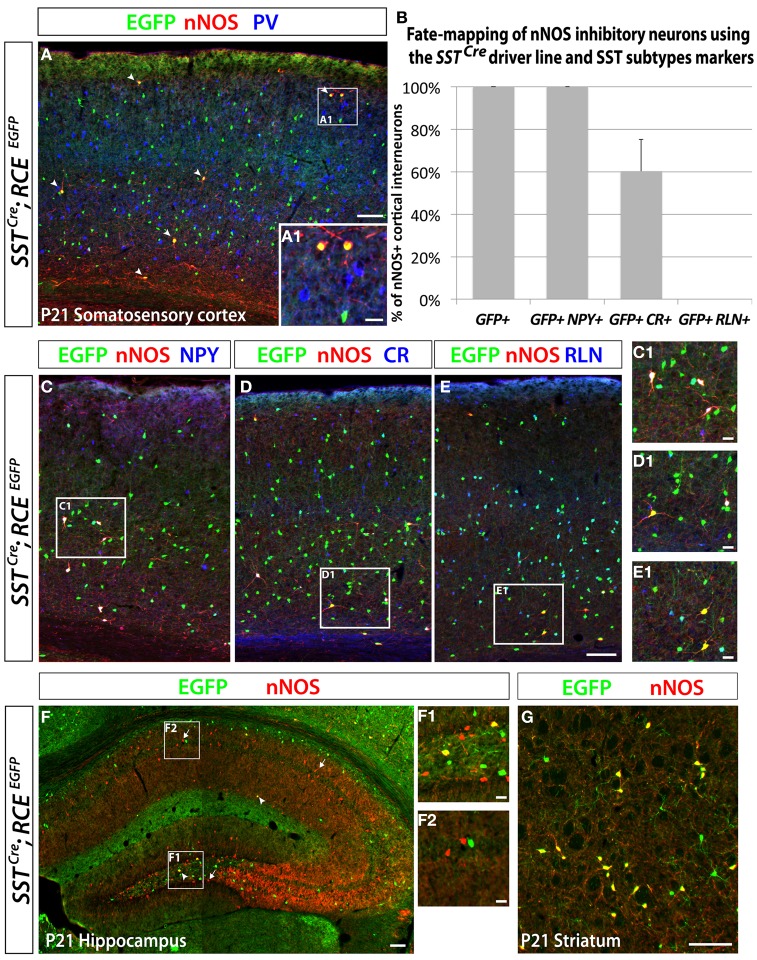
**Neocortical nNOS+ cells belong to the medial ganglionic eminence-derived somatostatin population of interneurons.** Use of the *SST*^*Cre*^ driver in combination with the RCE reporter allows the identification of the SST-expressing population of inhibitory neurons both in the neocortex and the hippocampus. MGE-derived interneurons represent ~70% of the entire interneuron population and express either somatostatin (SST) (~30%) or parvalbumin (PV) (~40%). Immunostaining using those two markers permits visualization of the entire population of inhibitory neurons that come from the MGE. **(A)** The entire number of nNOS cells (red) in *SST*^*Cre*^; *RCE*^*EGFP*^ P21 somatosensory cortex also expressed GFP (arrows) but not PV (blue only), either in deep or superficial (A1) layers. **(B–E)** Histograms and representative pictures of triple stainings performed on the *SST*^*Cre*^; *RCE*^*EGFP*^ P21 somatosensory cortex to determine the proportion of nNOS cells co-expressing neurochemical markers found in the SST-expressing population of inhibitory neurons, such as Neuropeptide Y (NPY), Calretinin (CR), Reelin (RLN). The entire neocortical nNOS cells population belongs to the MGE-derived SST-expressing population of GABAergic neurons and account for 4.1 ± 0.8% of it (data not show). Moreover, 100% of SST+/nNOS+ neurons co-express NPY, 60 ± 15% co-express CR, and none of them co-express RLN. **(F)** To determine if hippocampal nNOS neurons belong to the MGE-derived SST-expressing population of inhibitory neurons, we performed immunolabeling of nNOS and EGFP on *SST*^*Cre*^; *RCE*^*EGFP*^ P21 hippocampus. Only a small number of nNOS neurons express EGFP (arrowheads) in *SST*^*Cre*^; *RCE*^*EGFP*^ P21 hippocampus, thus revealing their inclusion in the SST-expressing population of MGE-derived interneurons. This scarce population, as compared to the majority of nNOS neurons that do not express GFP (arrows), is most consistently found in the dentate gyrus hilus (F1) (F1, hilar region and subgranular layer of the dentate gyrus; F2, CA1 stratum oriens, pyramidale and radiatum). **(G)** To determine if striatal nNOS neurons belong to the MGE-derived SST-expressing population of interneurons, we performed immunolabeling of nNOS and EGFP on *SST*^*Cre*^; *RCE*^*lEGFP*^ P21 striatum and revealed that about 93% of nNOS expressing striatal neurons belong the SST-expressing population. Data represent mean ± SEM (*n* = 3 independent brains). Scale bars correspond to 100 μm **(A,C–E,F,G)** and 25 μm (A1,C1–E1,F1,F2).

In order to eludicate the neurochemical profile of the dMGE-derived nNOS-expressing inhibitory neurons, we performed immunostaining for nNOS and SST, PV, NPY, CR, and RLN, which are characteristic markers of inhibitory neuron subtypes. Double immunostaining performed with the antibodies nNOS and SST or PV in the juvenile somatosensory mouse cortex (P21) shows that virtually all nNOS cells co-express SST but not PV (data not shown). A further genetic fate mapping analysis carried out using the *SST*^*Cre*^; *RCE*^*EGFP*^ line confirmed that all neocortical nNOS cells belong to the SST-expressing population, and none of them express PV (Figures 5A,B). In addition, we did triple labeling, to discern between the SST/CR and SST/RLN co-expressing populations versus the STT-negative/CR and /RLN expressing cells that are CGE-derived (Rudy et al., 2011). It is worth mentioning that NPY expression also seems to be shared by subpopulations of several different non-overlapping subtypes that have been shown to originate from the MGE, CGE, and POA (Fogarty et al., 2007; Xu et al., 2008; Gelman et al., 2009; Lee et al., 2010; Miyoshi et al., 2010). All the nNOS/SST double positive cells also co-express NPY (Figures 5B,C), 60 ± 15% express CR (Figures 5B,D), and none of the nNOS/SST double positive cells expressed RLN (Figures 5B,E). This is in contrast to the SST population in general, among which only around 25% co-express CR. This further supports a dorsal MGE origin for nNOS neocortical neurons since this population has been shown to also be enriched in SST/CR double positive interneurons (Sousa et al., 2009). In contrary, the majority of hippocampal nNOS cells do not express SST (Figure 5F), except for a small proportion of nNOS interneurons, mainly located in the dentate gyrus hilus (Figure 5F1). Interestingly, virtually all the striatal nNOS neurons also express SST (Figure 5G), once again suggesting that striatal, but not nNOS hippocampal neurons, are related to nNOS neocortical neurons in terms of origin and phenotype.

### The transcription factor *Sox6* is required for the specification of neocortical nNOS GABAergic neurons

**Figure 6:**
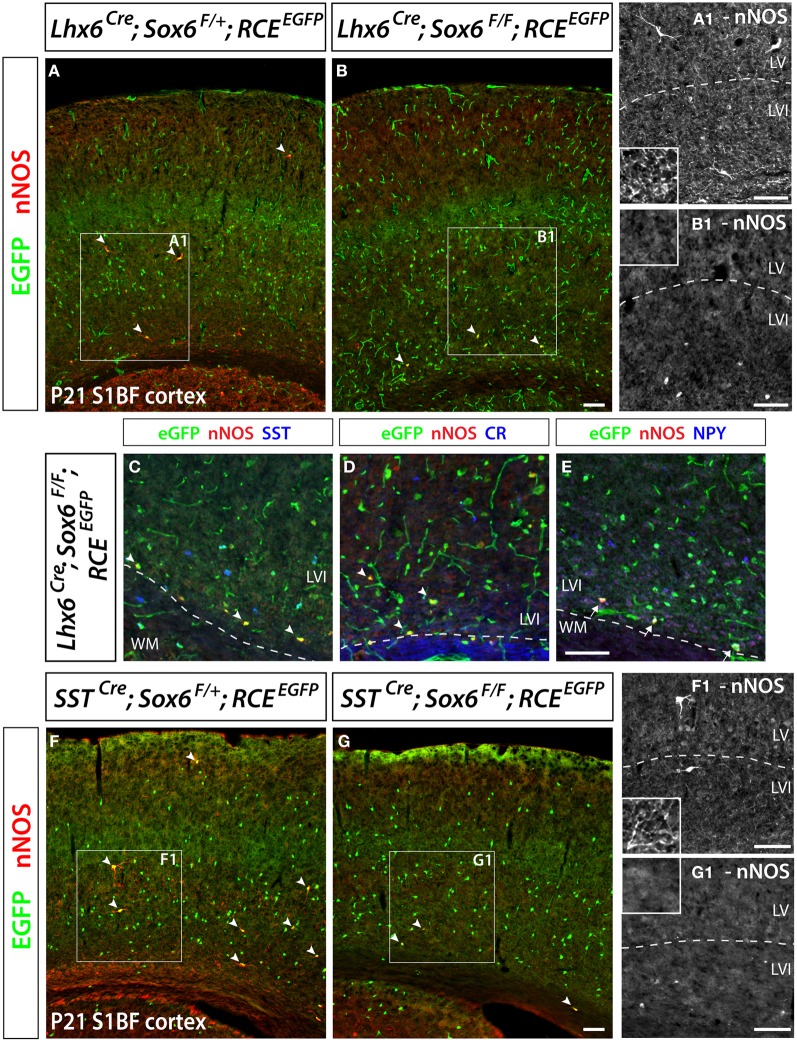
**Conditional removal of the transcription factor *Sox6* affects the nNOS-expressing population of neocortical GABAergic inhibitory neurons.** The *Lhx6*^*Cre*^ driver line is used to conditionally inactivate *Sox6* in MGE-derived GABAergic neurons and simultaneously fate map the recombined population. **(A,B)** The expression of nNOS and their axonal and dendritic arborization are affected within the population of nNOS+/GFP+ cells (arrowheads) in the P21 somatosensory mouse cortex of *Sox6* mutants (*Lhx6*^*Cre*^; *Sox6*^*F/F*^; *RCE*^*EGFP*^) (**B**–B1) vs. control (*Lhx6*^*Cre*^; *Sox6*^*F*/+^; *RCE*^*EGFP*^) (**A**–A1). Note the absence of nNOS-expressing neurons in superficial layers (II/III), the weaker intensity of nNOS staining in deep layers (V/VI) of *Sox6* mutants vs. control (compare A1 and B1), as well as the absence of nNOS-containing neurites spanning the entire cortical thickness in *Sox6* mutants vs. control (compare close-up insets in A1 and B1). **(C–E)** To determine if the removal of *Sox6* in nNOS cells affects their specification, we performed triple staining of EGFP, nNOS (red) in combination with SST, CR, or NPY (blue). This revealed that nNOS-expressing inhibitory neurons no longer express SST and CR in *Sox6* mutants (arrowheads) **(C,D)**. However, NPY expression is unaffected in *Sox6* mutants (arrows) **(E). (F,G)** To exclude any non-cell autonomous effect on the phenotype, we sought to determine if the *SST*^*Cre*^ removal of *Sox6* recapitulates the phenotype observed upon *Lhx6*^*Cre*^ removal of *Sox6*. EGFP/nNOS double staining of P21 somatosensory cortex revealed that MGE-derived SST/nNOS-expressing GABAergic neurons (arrowheads) are consistently affected by the loss of *Sox6*, as evidenced by the absence of nNOS cells in layer II/III, the decreased intensity of nNOS labeling in layer V/VI and the absence of nNOS+ neurites in the cortex. Data represent mean ± SEM (*n* = 3 independent brains for each genotype). Scale bars correspond to 100 μm.

We have previously shown that the transcription factor *Sox6* is present in most if not all MGE-derived neocortical inhibitory neurons (Batista-Brito et al., 2009). *Sox6* expression is dependent on the activity of the transcription factor *Lhx6*, which is specifically expressed in all MGE-derived interneurons (Du et al., 2008). We reported that conditionally removing *Sox6* in MGE interneurons leads to a 30% reduction of the number of neocortical neurons expressing SST, and a complete loss of SST/CR-expression in mutant cells. In the present study, to address if in *Sox6* mutants the nNOS subtype was among the affected SST population, we removed *Sox6* from neocortical MGE-derived interneurons while simultaneously fate mapping the cells. To this end we used a conditional allele of *Sox6* (*Sox6*^*F/F*^) (Dumitriu et al., 2006) crossed with the *Lhx6*^*Cre*^ driver line and the *RCE*^*EGFP*^ reporter line. The conditional mutant and control generated are *Sox6*^*F/F*^; *Lhx6*^*Cre*^; *RCE*^*EGFP*^ and *Sox6*^*F*/+^; *Lhx6*^*Cre*^; *RCE*^*EGFP*^, respectively. In *Sox6*^*F/F*^; *Lhx6*^*Cre*^; *RCE*^*EGFP*^ mutants we could still identify nNOS neurons, however, nNOS somatic expression appeared to be diminished relatively to control animals (Figures 6A,B). While the total number of neocortical nNOS neurons tends to be slightly decreased in control vs mutant (4.8 ± 1.8 nNOS+ cells/mm^2^ vs 3.4 ± 2.1 nNOS+ cells/mm^2^, respectively), nNOS cells were rarely found in the superficial layers of *Sox6* conditional mutants. We next asked if the neurochemical markers NPY, SST, and CR were affected in neocortical mutant nNOS neurons. The somatic nNOS staining, in combination with the EGFP expression of fate mapped cells allowed for the identification of *Sox6* mutant MGE-derived inhibitory neurons. *Sox6* mutant nNOS neurons no longer express SST (Figure 6C) or CR (Figure 6D), but do retain the expression of NPY (Figure 6E). While cortical nNOS expressing neurons are affected by the conditional loss of *Sox6*, their hippocampal counterparts remain largely unaffected in the conditional *Sox6* mutants (data not shown). In agreement to what we reported previously (Batista-Brito et al., 2009), we observed a non-cell autonomous increase in the number of cells expressing NPY, as well as a diffuse and non-cell autonomous increase in the background of nNOS staining (data not shown). Furthermore, the cell morphology of mutant nNOS cells seemed to be severely affected, with these cells possessing considerably less dendritic and axonic arborization (compare Figures 6A1 and 6B1). However, it is not clear if this represents an alteration in the morphology of the cells, or if it is the result of weaker immunostaining. A more careful characterization, such as filling the mutant cells with a dye will be necessary to resolve this issue. However, since this cell population is so sparse, we did not succeed in identifying nNOS cells for detailed morphological analysis.

**Figure 7:**
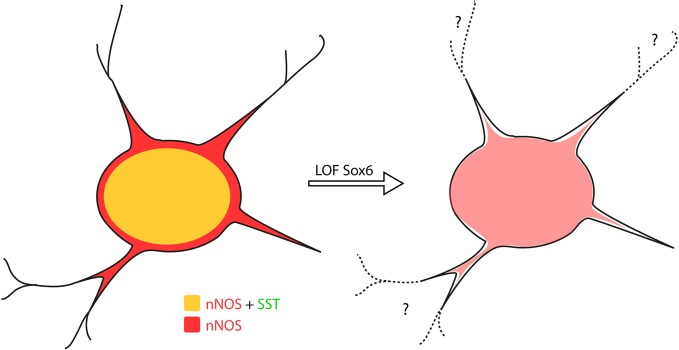
***Sox6* loss-of-function phenotype in nNOS neocortical inhibitory neurons.** Removal of *Sox6* in nNOS neocortical inhibitory neurons leads to the absence of SST expression, decreased levels of nNOS in the soma and absence of nNOS-expression in neurites, which are no longer visible. It remains to be determined whether the inability to detect neurites is caused by their truncation, or due to weak nNOS-expression.

As previously described, *Sox6*^*F/F*^; *Lhx6*^*Cre*^; *RCE*^*EGFP*^ mutants develop generalized seizures by P16 followed by death of the animals between P17 and P19 from prolonged seizures, most likely due to a severe loss of inhibition from the affected PV-expressing basket cells (Batista-Brito et al., 2009). To test if the phenotype observed in nNOS cells was a non-cell autonomous consequence of seizure activity, we generated the mutant mice, *Sox6*^*F/F*^; *SST*^*Cre*^; *RCE*^*EGFP*^, in which *Sox6* is only removed in SST-expressing MGE-derived inhibitory neurons, leaving the PV interneurons unaffected. *Sox6*^*F/F*^; *SST*^*Cre*^; *RCE*^*EGFP*^ mice appear to be phenotypically normal and do not show premature death nor develop obvious seizures. We confirmed that similarly to what we observed in *Sox6*^*F/F*^; *Lhx6*^*Cre*^; *RCE*^*EGFP*^ mutants, nNOS neocortical neurons in *Sox6*^*F/F*^; *SST*^*Cre*^; *RCE*^*EGFP*^ animals still fail to express SST and CR (data not shown) and have reduced nNOS expression at the soma level (Figures 6F,G), as well as seemingly less elaborated arborization of their processes (Figures 6F1,G1). Our results suggest that the transcription factor *Sox6* is necessary for the proper specification of nNOS neocortical neurons, and possibly necessary for the development of complex arborization of their processes (Figure 7).

## Discussion

In the present study, we characterized the contribution of nNOS-expressing cells in cortical GABAergic populations, as well as their place of origin and genes involved in their development. We show that contrary to the hippocampus, where nNOS interneurons are a numerous GABAergic class, nNOS (type I) neurons in the neocortex only constitute a very small portion of the GABAergic population. In accordance with previous studies, we observed that the majority of hippocampal nNOS GABAergic neurons originate within the MGE (Tricoire et al., 2010, 2011), however, a considerable percentage of this neuronal type also arises from the more ventral structures CGE and/ or POA. In contrast, the vast majority of neocortical nNOS GABAergic neurons are produced in the MGE, mainly in the dMGE. Hippocampal nNOS GABAergic neurons are also more diverse than their neocortical counterparts with regards to their molecular heterogenity. Neocortical nNOS cells appear to be a rather homogeneous population consisting perhaps of only two sub-populations. All nNOS-expressing neocortical inhibitory neurons express SST and NPY while 60% of them express CR. However, hippocampal nNOS GABAergic neurons express a variety of molecular markers such as NPY, SST, VIP, CR (Tricoire et al., 2010), and can further be subdivided on neurogliaform cells (NGC) and Ivy cells (IvC) accordingly to their morphology and the laminar location on their soma (Fuentealba et al., 2008). Finally we show that while neocortical nNOS cells require the transcription factor *Sox6* in order to develop their mature morphology and express characteristic molecular markers, the maturation of the hippocampal nNOS cells appears to be unaffected by *Sox6* loss of function. This again strengthens the evidence supporting that nNOS GABAergic neurons in these two distinct, but highly related cortical structures, are generated through independent developmental programs. Interestingly neocortical nNOS neurons appear to be more closely related to their striatal counterparts.

### Neocortical nNOS GABAergic neurons mainly originate in the dorsal MGE

**Figure 8:**
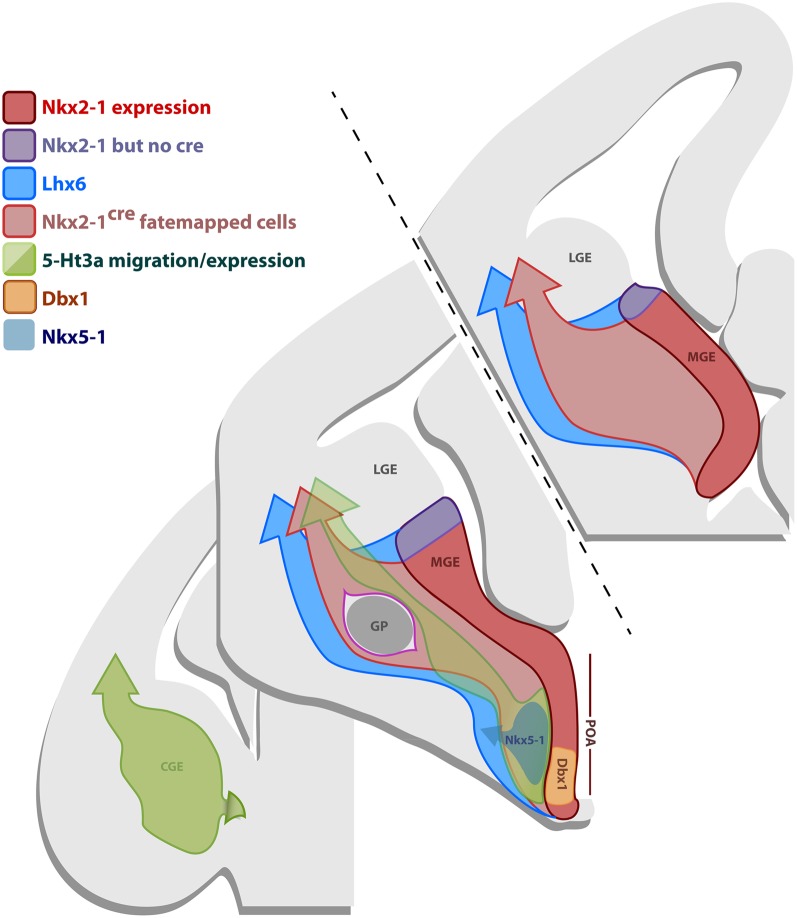
**Gene expression in the subpallial proliferative regions.** Schematic representation of coronal sections of an E12.5 brain illustrating the gene expression profile of the progenitor regions MGE, CGE and POA. The most frontal section in the anterior/posterior axis (upper-left section) only comprises the MGE, the middle section contains the MGE, CGE and the POA, while the most caudal section (lower-right section) only shows the CGE. The MGE expresses Nkx2.1 (red domain), however, the BAC line *Nkx2.1*^*Cre*^ expresses the Cre recombinase in most of the MGE, except for its most dorsal part (dMGE) (purple domain). Nkx2.1 and Cre in the line *Nkx2.1*^*Cre*^ are also expressed in some of the POA. Consequently, fate mapping with the line *Nkx2.1*^*Cre*^ labels cells originating from the majority of the MGE, except for the dMGE (purple domain), and some of the POA, that migrate into the dorsal telencephalon (light-red arrow). *Lhx6* is downstream of *Nkx2.1* and is expressed in postmitotic neurons originating from the MGE and part of the POA. Fate mapping with the *Lhx6*^*Cre*^ line labels cells derived from the MGE (including dMGE-purple domain) and part of the POA that migrate into the dorsal telencephalon (blue arrow). The CGE and part of the POA express *5-Ht3a* (green domain). Fate mapping with the BAC line *5-Ht3a^*EGFP*^* labels cells arising from the CGE and part of the POA that migrate into the dorsal thelencephalon (green arrow). Specific domains of the POA also express *Nkx5.1* (gray) and *Dbx1* (orange).

Neocortical GABAergic neurons arise from three telencephalic transient embryonic structures, the MGE, CGE, and POA (Batista-Brito and Fishell, 2009). In Figure 8, we show a schematic of genes expressed in those regions, which are important for the production and specification of neocortical and hippocampal GABAergic neurons. The MGE expresses *Nkx2.1* (Sussel et al., 1999) (red domain in Figure 8). In order to fate map the MGE we used the BAC line *Nkx2.1*^*Cre*^, in which Cre expression is driven by the *Nkx2.1* promoter (Xu et al., 2008). While Nkx2.1 protein is expressed throughout the MGE and POA, Cre expression in the *Nkx2.1*^*Cre*^ driver spares the most dorsal part of the MGE (dMGE) (purple domain in Figure 8) (Xu et al., 2008). The absence of Cre expression in the dMGE of this BAC transgenic line might be due to positional effects or the absence of a critical part of the *Nkx2.1* promoter. However, because the expression of *Nkx2.1* mRNA is weaker in the dMGE than the rest of the MGE (Sussel et al., 1999), it has been suggested that the absence of Cre expression in the dMGE reflects a weaker *Nkx2.1* gene activation in this domain (Xu et al., 2008). The fact that the expression of another independently produced *Nkx2.1*^*Cre*^ BAC line is also absent from the dMGE (Kessaris et al., 2006; Fogarty et al., 2007) further supports this idea. Fate mapping of the totality of the MGE can be accomplished by using the BAC transgenic line *Lhx6*^*Cre*^ (Fogarty et al., 2007). *Lhx6* is expressed in postmitotic neurons derived from the MGE, as well as the POA (blue domain in Figure 8) (Fogarty et al., 2007; Du et al., 2008).

The most significant insight into the developmental origin of neocortical nNOS GABAergic was provided by the comparison between the fate mapping of *Nkx2.1*- and *Lhx6*-derived lineages. A minority of the neocortical nNOS cells (about 30%) could be accounted by *Nkx2.1* fate mapping, but virtually all of the neocortical nNOS cells could be accounted by *Lhx6* fate mapping (about 95%). Since the only progenitor region labeled by *Lhx6* but not *Nkx2.1*- fate mapping is the dMGE, our data strongly suggests that the dMGE is the source of the majority of neocortical nNOS neurons.

In our experience, no nNOS-positive neocortical neurons were labeled by the *5-Ht3a^*EGFP*^* line, which labels the CGE and possibly part of the POA (Lee et al., 2010). Given that previous studies have shown that a small fraction of the cortical nNOS-expressing cortical neurons originate in the *Dbx1* domain of the POA (Gelman et al., 2009, 2011), we conclude that the *5-Ht3a^*EGFP*^* BAC transgenic allele is unlikely expressed in the *Dbx1*-positive domain within the POA. This is further supported by the presence of markers typical for MGE (and thus not *5-Ht3a^*EGFP*^*) such as PV and SST in the *Dbx1*-fate mapped population.

### Diverse origins of nNOS neocortical and hippocampal GABAergic neurons and the use of neurochemical markers

Fate mapping studies have shown that the neocortical interneuron subtypes arising from the MGE and CGE are, for the most part, mutually exclusive (Rudy et al., 2011). For instance the hippocampal and neocortical PV-expressing fast spiking basket cells are solely produced in the MGE (Butt et al., 2005; Tricoire et al., 2011), while the CGE is the source of the VIP-expressing bipolar interneuron populations (Butt et al., 2005; Lee et al., 2010; Tricoire et al., 2011). This suggests that interneuron precursors are subjected to distinct genetic programs initiated within the eminences that at least in part, determine their subtype identity. However, some molecular markers, such as the neurotransmitter NPY, are not specific for a particular interneuron subtype. Both neocortical and hippocampal NPY-expressing inhibitory neurons comprise a wide variety of subtypes (Karagiannis et al., 2009; Tricoire et al., 2011) and are derived from the MGE, CGE, and POA (Fogarty et al., 2007; Gelman et al., 2009; Lee et al., 2010; Tricoire et al., 2011). Furthermore, the level of expression of NPY is highly dependent on neuronal activity (Sperk et al., 1992; Schwarzer et al., 1995; Baraban et al., 1997). Taken together, these observations suggest that NPY should be used with caution as a marker of subtype identity.

Here we show that similar to PV neurons, neocortical nNOS cells are solely derived from the MGE and constitute a rather homogeneous subset of long range projections SST-expressing neurons (Tomioka et al., 2005), therefore, suggesting that in the neocortex nNOS might be a good indicator of neuronal subtype. By contrast, hippocampal nNOS cells are produced from a number of subpallial regions, have different morphologies and express a variety of neurochemical markers (Tricoire et al., 2010, 2011). This suggests that using nNOS as a subtype marker must be done carefully, especially when comparing structures. Moreover, the comparison between subtype diversity of hippocampal and neocortical cells expressing nNOS suggests that the location of birth of nNOS neurons is not the sole factor determining their marker expression, and that environmental cues present in the neocortex and hippocampus likely influence the expression of nNOS. It is likely that, except for nNOS expression, neocortical and hippocampal nNOS neurons are unrelated, and involved in different functions. Interestingly, neocortical nNOS neurons appear to have more in common with their counterparts in the striatum. Although being the largest striatal subtype, nNOS neurons are largely derived from the *Nkx2.1* domain and similar to the cortex express both SST and NPY.

### The transcription factor *Sox6* is required for the specification of neocortical nNOS GABAergic neurons

Previous work has shown that loss of function of Nkx2.1 at the progenitor level results in a decreased number of nNOS-expressing neurons migrating tangentially into the cortex during development (Anderson et al., 2001), and a vast reduction of nNOS interneurons in the hippocampus at adult ages (Tricoire et al., 2010). We have previously shown that loss of the transcription factor Sox6, a gene downstream of Nkx2.1 and Lhx6 affects the radial migration and maturation of PV- and to a lesser extent SST-expressing neocortical inhibitory neurons. PV expression in fast-spiking basket cells is by and large lost in *Sox6* mutants while the effect on SST expression is much more modest. One SST-positive population that is affected by the loss of *Sox6* is the SST/CR double positive population where CR expression is lost completely (Batista-Brito et al., 2009). Given that in the neocortex nNOS neurons constitute a subpopulation of SST-neurons, we investigated whether the neurochemical characteristics, number, positioning or morphology of nNOS cells would be affected when *Sox6* is conditionally removed in Lhx6 expressing cells. Removal of *Sox6* had a marked effect on nNOS expressing neocortical inhibitory neurons. While nNOS persists (at low but detectable levels), *Sox6* mutant nNOS-expressing cells no longer express SST or CR, and are possibly less numerous, as indicated by the fact that in mutant animals nNOS neocortical neurons tend to be absent from the superficial layers. Furthermore, it appears based on nNOS staining, that mutant nNOS neurons display underdeveloped arborization of their processes. This contrasts with our previous results showing that even though *Sox6* mutant PV cells are immature, they still develop normal morphology (Batista-Brito et al., 2009). Taken together, our data show that removal of *Sox6* in nNOS neocortical neurons compromises their specification and also perhaps their morphology. Since the phenotype of nNOS mutant cells remained the same when *Sox6* was selectively removed in SST-expressing neurons, we argue that the effect is cell autonomous, or at least not caused by disrupted cortical network activity associated with PV-expressing fast spiking interneuron dysfunction. Similar to the neocortex, nNOS cells deprived of *Sox6* gene function in the striatum have reduced SST staining (data not shown). However, we did not detect any obvious defects in hippocampal nNOS interneurons in *Sox6* mutants (data not shown).

### Functional role of nNOS GABAergic neurons

In the hippocampus (Jinno and Kosaka, 2002b), and likely in the neocortex, nNOS-expressing GABAergic neurons are the major source of nitric oxide (NO). NO regulates synaptic plasticity of glutamatergic and GABAergic synapses (Garthwaite and Boulton, 1995; Arancio et al., 1996; Burette et al., 2002; Szabadits et al., 2007; Garthwaite, 2008), and in the hippocampus potentiates GABA release (Li et al., 2002; Yu and Eldred, 2005). Even though neocortical nNOS neurons are distinct, and possibly differently regulated than their hippocampal counterparts, nNOS cells in the neocortex also function to control the level of GABA release and synaptic plasticity (Hardingham and Fox, 2006; Dachtler et al., 2011). While the number of nNOS neurons in the neocortex is small, their impact on the neocortical network through NO release might be significant as they are highly ramified, and project long distances rostro-caudally and medio-laterally, connecting neocortical areas up to 6–8 mm apart (Tomioka et al., 2005). In fact, it has been recently shown that specifically damaging nNOS neurons in the neocortex results in dysinhibition of the entire neocortical network and strong alterations of the spatio-temporal dynamics of the neocortex (Shlosberg et al., 2012). Cortical nNOS expressing neurons are also implicated in regulating local blood flow by transforming neuronal signals into vascular responses (Cauli et al., 2004), suggesting that neurogliaform-like neurons that express nNOS are likely to mediate cortical vasodilatations and/or vasoconstrictions that happen *in vivo* (Karagiannis et al., 2009). Moreover, it has also been shown that nNOS neurons are active during sleep (Gerashchenko et al., 2008). It has been reported that the functional activation of nNOS neurons during sleep is restricted to the cortex (Pasumarthi et al., 2010), suggesting that neocortical nNOS are major players in homeostatic sleep regulation (Gerashchenko et al., 2008; Pasumarthi et al., 2010). Importantly, while the regulation of the levels of NO is critical, an excessive production of NO can lead to neurotoxicity (Dawson and Dawson, 1998), suggesting that the nNOS population in addition to normal brain function can result in disease states when perturbed.

In the future, in order to understand the contribution of nNOS expressing inhibitory neurons in the neocortical circuit, it would be interesting to investigate the intrinsic properties of nNOS neocortical neurons, as well as their synaptic inputs and outputs. However, in order to address these questions in such a sparse neuronal type, it will be necessary to specifically target nNOS cells, a goal that is achievable using recently developed genetic tools, such as the inducible Cre-diver line, *nNOS*^*creER*^ (Taniguchi et al., 2011). It would be particularly interesting to compare the electrophysiological properties of nNOS neocortical neurons to those of other SST expressing cells. Our hypothesis is that similar to nNOS cells in the striatum (Kawaguchi et al., 1995; Centonze et al., 2002) and SST neocortical interneurons (Kawaguchi and Kubota, 1996), nNOS cells in the neocortex will exhibit low threshold-spiking characteristics. However, due to the very different range of their output domains, we suspect that their functional roles in the network may be very different from those of SST^+^/nNOS^−^ neocortical interneurons. To address the functional role of neocortical nNOS neurons at both synaptic and circuit levels, it would be very interesting to selectively activate and suppress their activity using genetic methods such as opto- or pharmaco-genetics.

### Conflict of interest statement

The authors declare that the research was conducted in the absence of any commercial or financial relationships that could be construed as a potential conflict of interest.
